# Distinct and synergistic feedforward inhibition of pyramidal cells by basket and bistratified interneurons

**DOI:** 10.3389/fncel.2015.00439

**Published:** 2015-11-05

**Authors:** Michele Ferrante, Giorgio A. Ascoli

**Affiliations:** ^1^Computational Neurophysiology Laboratory, Center for Memory and Brain, Psychology Department, Boston UniversityBoston, MA, USA; ^2^Krasnow Institute for Advanced Study, George Mason UniversityFairfax, VA, USA

**Keywords:** feedforward inhibiton, bistratified, basket, interneurons, CA1 pyramidal cells, neuronal connectivity, hippocampus, input-output transformation

## Abstract

Feedforward inhibition (FFI) enables pyramidal cells in area CA1 of the hippocampus (CA1PCs) to remain easily excitable while faithfully representing a broad range of excitatory inputs without quickly saturating. Despite the cortical ubiquity of FFI, its specific function is not completely understood. FFI in CA1PCs is mediated by two physiologically and morphologically distinct GABAergic interneurons: fast-spiking, perisomatic-targeting basket cells and regular-spiking, dendritic-targeting bistratified cells. These two FFI pathways might create layer-specific computational sub-domains within the same CA1PC, but teasing apart their specific contributions remains experimentally challenging. We implemented a biophysically realistic model of CA1PCs using 40 digitally reconstructed morphologies and constraining synaptic numbers, locations, amplitude, and kinetics with available experimental data. First, we validated the model by reproducing the known combined basket and bistratified FFI of CA1PCs at the population level. We then analyzed how the two interneuron types independently affected the CA1PC spike probability and timing as a function of inhibitory strength. Separate FFI by basket and bistratified respectively modulated CA1PC threshold and gain. Concomitant FFI by both interneuron types synergistically extended the dynamic range of CA1PCs by buffering their spiking response to excitatory stimulation. These results suggest testable hypotheses on the precise effects of GABAergic diversity on cortical computation.

## Introduction

CA1 Pyramidal Cells (CA1PCs) constitute the output of the hippocampal tri-synaptic circuit, relaying the information processed by area CA3 onto the subiculum and the deep layers of the entorhinal cortex. CA1PCs activity encodes spatial (O’Keefe and Dostrovsky, 1971) and temporal (MacDonald et al., 2011) features of episodic memories. This representation is mediated by the integration of excitatory and inhibitory inputs from ~30,000 glutamatergic and ~1700 GABAergic synapses, respectively (Megías et al., 2001). CA1PCs receive widely divergent and convergent stimulations from the ipsilateral and contralateral Schaffer collaterals. Without inhibitory control, even minimal alterations in the number of activated pre-synaptic neurons could result in all-or-none recruitment of the whole CA1PC population (Shadlen and Newsome, 1998). To counteract the substantial activity fluctuations of CA3 pyramidal cells (Wilson and McNaughton, 1993; Csicsvari et al., 2000), CA1PCs use feedforward inhibition (FFI) to expand the dynamic range of stimulus strengths over which they faithfully respond (Pouille et al., 2009). FFI is a ubiquitous connectivity motif in hippocampus and neocortex in which an axonal pathway (e.g., Schaffer collateral from CA3) excites both the principal cells in an area (CA1PCs) and a group of GABAergic interneurons that contact the same target (Buzsáki, 1984). FFI also allows CA1PC dendrites to sum incoming activity over broader time windows while enforcing precise coincidence detection in the soma (Pouille and Scanziani, 2001). This mechanism increases the temporal fidelity of the circuit by reducing spike onset jitter.

Synaptic contacts in CA1PCs are organized in orderly spatial sub-domains along complex dendritic trees enriched with diverse sets of active properties. In particular, two physiologically, biochemically, and morphologically distinct interneuron classes can inhibit CA1PCs in a feedforward manner (Buhl et al., 1996; Halasy et al., 1996; Klausberger, 2009; Tricoire et al., 2011): basket cells are fast-spiking, express parvalbumin, and target CA1PCs perisomatically in stratum pyramidale; bistratified cells are regular-spiking, express 5HT3R, NPY, SOM, and Coup-TFII (all of which are absent in basket cells), and target CA1PCs on the basal dendrites in stratum oriens and on the apical dendrites in stratum radiatum^1^ (Wheeler et al., 2015). Therefore, these two FFI pathways can in principle form distinct layer-specific computational sub-domains within the same CA1PC. Basket and bistratified cells in the CA1 area are activated by the same (CA3 Schaffer collateral) axons in a feedforward manner, but the EPSP dynamics and kinetics in these two cell types are different (Buhl et al., 1996). Moreover, basket and bistratified interneurons exhibit clearly distinct intrinsic and computational properties. For instance, compared to basket cells, bistratified interneurons have a more hyperpolarized resting membrane potential (−64.5 vs. −69.2 mV) and a nearly double input resistance (31.3 vs. 60.2 MΩ).

Due to their differences in intrinsic excitability and network connectivity, basket and bistratified cells might differentially affect CA1PCs activity. Moreover, their combined action might produce non-trivial synergistic effects on the computational properties of CA1PCs. Despite ongoing efforts to quantitatively characterize the CA1 circuit (Bezaire and Soltesz, 2013), the distinct functional contributions of different interneuron types on CA1PCs remain technically challenging to tease apart in the wet lab. The present study investigates the specific effects of basket and bistratified FFI on CA1PC using biophysically and morphologically detailed computational simulations constrained by and validated against experimental data. Specifically, we analyzed the CA1PC population activity as well as the single neuron spike probability and onset in four conditions: FFI by both basket and bistratified cells; FFI by basket cells alone; FFI by bistratified cells alone; and pure excitation. Furthermore, we investigated how modulating the synaptic strength of the two interneuron populations may regulate CA1PC firing.

## Materials and Methods

**Figure 1:**
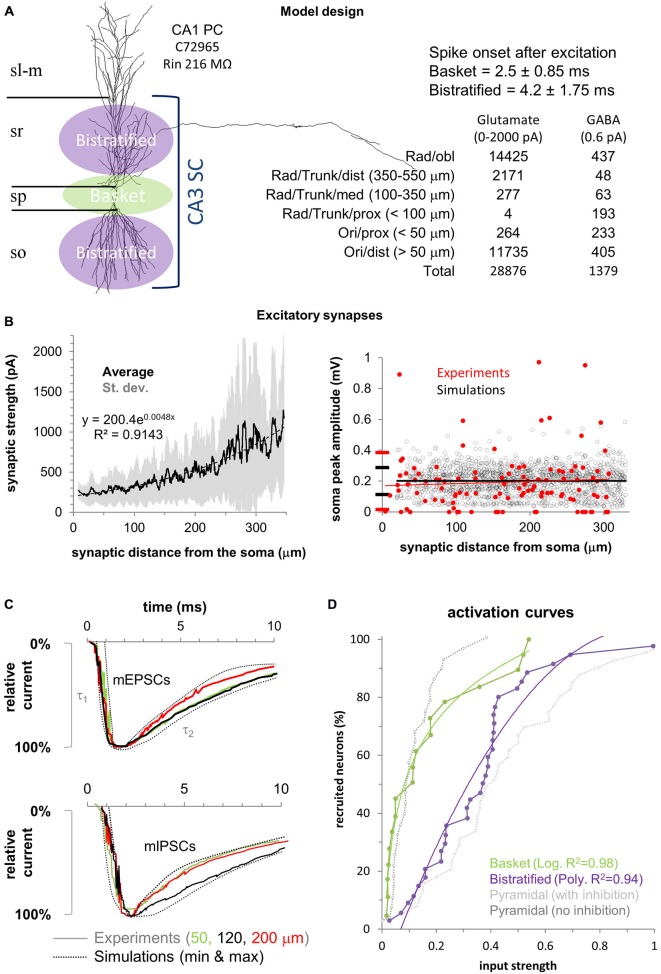
**Model design and experimental validation. (A)** *Left*: Schematic of the feedforward inhibition (FFI) model in a CA1 pyramidal cell (CA1PC) illustrated with one of the 40 3D morphologies used. Basket cells synapse on the CA1PC perisomatic region (green) while bistratified cells synapse on the apical and basal dendrites (purple). *Right*: Temporal activation of the two interneuron populations and number of excitatory and inhibitory synapses with their respective spatial distributions. Stratum lacunosum-moleculare (sl-m), stratum radiatum (sr), stratum pyramidale (sp), and stratum oriens (so). **(B)*** Left*: Synaptic current increase (synaptic scaling) along the proximal-to-distal axis in the 40 neuronal morphologies. Solid black line shows the average synaptic strength for all synapses, with standard deviation in gray. Black dotted line is the exponential fit (equation on the chart). *Right*: Synaptic normalization at the soma compared to available experimental measurements (in red). **(C)** The model (dotted lines) replicates the experimental (solid traces) rise (τ_1_), and decay (τ_2_) time constants for mEPSCs (top panel) and mIPSCs (bottom panel) at different distances from the soma (color coded). **(D)** Fitting used in the model for the activation curves of the two interneuron populations (data from Pouille et al., 2009). Experimental activation curves for CA1PCs with (gray) and without (black) inhibition are provided for reference (respectively “control” and “gabazine” conditions from Pouille et al., 2009).

A biophysically realistic model of FFI in CA1PCs (Figure 1A) was designed based on previous work (Li and Ascoli, 2006; Ferrante et al., 2009). Model and simulations were implemented in NEURON (Hines and Carnevale, 1997) v7.3 using variable time step on a 32-bit Pentium quad-core Dell precision T3500 running Windows 7. The model is publicly available under the ModelDB section of SenseLab^2^.

### Neuronal Morphologies and Membrane Properties

The model included 40 digitally reconstructed CA1PC morphologies downloaded from the Korte (Michaelsen et al., 2010), Claiborne (Carnevale et al., 1997), Amaral (Ishizuka et al., 1995), Turner (Pyapali et al., 1998), Larkman (Bannister and Larkman, 1995), Gulyás (Megías et al., 2001), and Spruston (Golding et al., 2005) archives of NeuroMorpho.Org (Ascoli et al., 2007). CVAPP (Cannon et al., 1998) was used to differentially tag oblique dendrites (in stratum radiatum) from the main apical trunk and distal branches (in stratum lacunosum-moleculare). Basal dendrites (in stratum oriens) were already pre-tagged in NeuroMorpho.Org.

Active (*I*_Na_, *I*_Kdr_, *I*_KA_, *I*_h_) and passive properties (*τ*_m_ = 28 ms, *R*_m_ = 28 kΩ · cm^2^, *R*_a_ = 50 Ω · cm) were the same for each neuronal morphology and have been previously described and experimentally validated for CA1PCs (Migliore et al., 2004, 2005; Ferrante et al., 2013). Briefly, *I*_Na_ and *I*_Kdr_ were uniformly distributed throughout the neuronal membrane (*g*_Na_ = 0.25 nS/μm^2^; *g*_Kdr_ = 0.1 nS/μm^2^), while *I*_KA_ and *I*_h_ increased linearly with the distance from the soma as in previously reported experiments (Hoffman et al., 1997; Magee, 1998), namely *g*_KA_ = 0.3 · (1 + dist/100) and *g*_h_ = 0.0005 · (1 + 3·dist/100).

As it can be appreciated from publicly available morphological tracings in NeuroMorpho.Org (Ascoli et al., 2007), the axons of the bistratified interneurons (NMO_ID: 02343, 02344, 02346, 02349 from Cossart et al., 2006) tend to selectively target stratum oriens and stratum radiatum, avoiding stratum pyramidale. In contrast, axons from basket cells (NMO_ID: 07323, 07326, 07338, 07339 from Glickfeld and Scanziani, 2006) tend to preferentially target the perisomatic region of CA1PCs (i.e., stratum pyramidale). This is consistent with seminal summaries clearly describing the morphologies and synaptic connectivity from bistratified (Somogyi and Klausberger, 2005) and basket (Buhl et al., 1994) cells to CA1PCs.

### Synaptic Properties

A realistic number (Megías et al., 2001) of excitatory (*n* = 28,876) and inhibitory (*n* = 1379) synapses were randomly redistributed in each simulation within spatial boundaries (Figure 1) defined to emulate available experimental data (Megías et al., 2001). The model assumes that all excitatory synapses from CA3 Schaffer collaterals are located in strata oriens and radiatum, while inhibitory synapses from basket and bistratified interneurons are spatially non-overlapping: basket cells synapse on the soma, proximal basal dendrites (<50 μm from the soma), and proximal apical dendrites (<100 μm from the soma). Bistratified cells target the dendrites more distally on both the basal (>50 μm from the soma) and apical arbors (>100 μm and up to 550 μm from the soma). The numbers and dendritic distributions of excitatory and inhibitory synapses allocated in each simulation are reported in Figure 1A.

In agreement with experimental results (Magee and Cook, 2000), the weight of each excitatory synapse was adjusted so as to yield an average somatic depolarization of 0.2 mV in all CA1PCs (Figure 1B). To achieve this, we placed an excitatory synapse in each compartment of the main trunk of every neuron (up to 320 μm from the soma, as in the experiments). The synaptic weights varied with the distance from the soma according to the same formula for all neurons: Syn weight = (A*dist^2^) + B. We then adjusted the parameters (*A* and *B*) so that the average somatic depolarization was 0.2 mV and did not depend on the synaptic distance from the soma (Li and Ascoli, 2006). Experimental data suggest that each CA1PC receives on average 11 synaptic contacts from both basket and bistratified cells (Bezaire and Soltesz, 2013) and their compound effect ranges between 5 and 25 pA (Andrásfalvy and Mody, 2006). The conductance of all inhibitory synapses was accordingly set to 0.6 pA.

The kinetics and reversal potentials for both excitatory and inhibitory synaptic currents (Figure 1C) were modeled by fitting double-exponential functions (*Exp2Syn*) to experimental voltage data (Andrásfalvy and Mody, 2006). For the excitatory synapses, rise and decay times were 0.5 and 5.5 ms, respectively, and the reversal potential was 0 mV. Local mIPSCs recordings (Andrásfalvy and Mody, 2006) reveal no variation of kinetics with distance from the soma. Thus, the fitted synaptic properties were identical for basket and bistratified cells: rise time 0.73 ms, decay 6.5 ms, and reversal potential -80 mV.

Synapses were activated asynchronously and each synapse was only activated once per simulation. The activation time for excitatory synapses was sampled from a Gaussian distribution with mean equal 5 ms and standard deviation equal to 2.34 ms. The model assumes that, due to the local nature of basket and bistratified inhibition, the spike transmission through the short axons adds a negligible temporal delay to synaptic onset relative the somatic firing of the presynaptic interneurons. Accordingly, in agreement with experimental evidence (Pouille et al., 2009), basket cell synapses were activated on average 2.5 ms after stimulation (standard deviation 0.92 ms) and bistratified synapses 4.2 ms after excitation (standard deviation 1.32 ms).

### Stimulation Protocol

To simulate varying stimulus strength, we increased the number of activated excitatory synapses one at the time, starting from 1. The outcome of every simulation for a given CA1PC was either a spike or not. For each given number of synapses, we run 50 simulations with every CA1PC. A CA1PC is considered to be “recruited” if it spikes in at least half of the simulations (≥25/50). In order to most meaningfully compare simulation with experimental data, we define a unitary input strength (following Pouille et al., 2009) as the number of activated excitatory synapses sufficient to recruit 95% of the CA1PCs (38 out of 40) in the presence of basket and bistratified FFI (“control” condition in Pouille et al., 2009). The activity of the two populations of interneurons in response to stimulation (that is, the proportion of activated inhibitory synapses) was simulated by using mathematical fitting (Figure 1D) that closely replicated (*R*^2^ > 0.94) the experimental data (Pouille et al., 2009). For each neuron, we stopped increasing the stimulus strength and ended the simulations when a spike was observed in all 50 stimulations (100% spike probability) for the last three numbers of activated synapses.

## Results

### Model Validation and CA1PC Population

**Figure 2:**
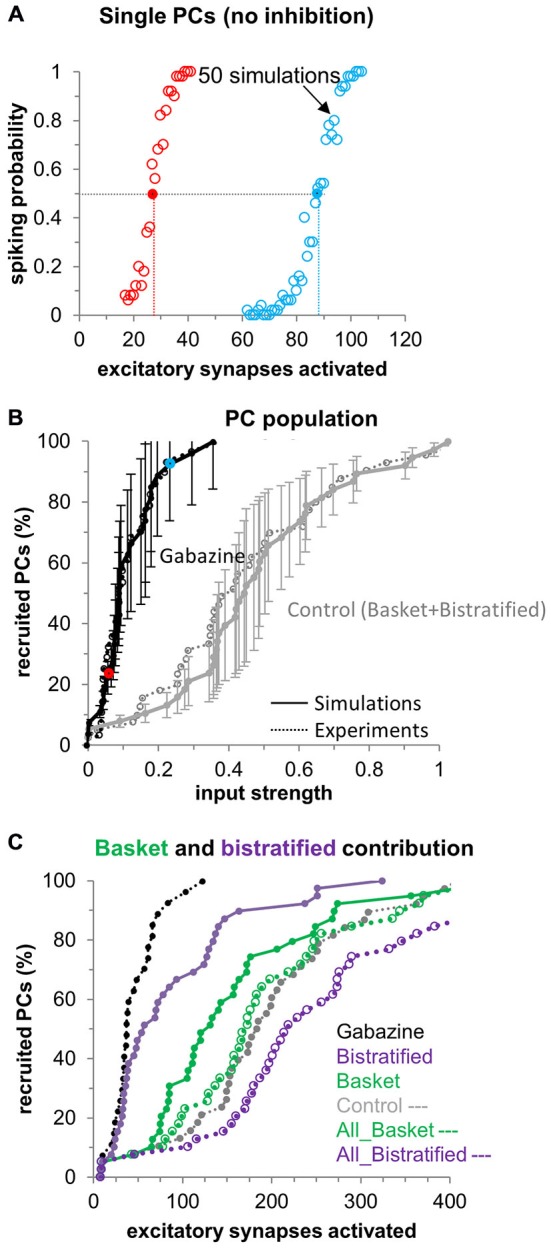
**CA1PC input/output curves: distinct FFI by basket and bistratified interneurons. (A)** CA1PC input/output curves for two representative neurons in the absence of inhibition. Each dot represents the spiking probability over 50 simulations each with random redistribution of the synaptic spatial location. The dotted lines indicate the number of synapses necessary to “recruit” the cell (i.e., passing the 50% spiking probability). **(B)** Activation curve of the CA1PC population (*n* = 40 cells). Simulations (solid lines with error bars) closely match experiments (dotted lines, from Pouille et al., 2009) for the control (black) and gabazine conditions (gray). Red and blue dots represent the cells shown in **(A)**. **(C)** Distinct contribution of basket (solid green line) and bistratified (solid purple line) interneurons to the CA1PC activation curve, and predicted effect if all the existent inhibitory synapses belonged to basket (dotted green line) or bistratified (dotted purple line) cells.

Despite identical distributions of active and passive properties, the natural diversity of CA1PC morphologies results in clear differences in excitability as evidenced by the input/output curves of two representative neurons (Figure 2A), and reflecting a similar variability in the experimental data (Pouille et al., 2009). The number of synapses necessary to recruit a CA1PC (that is, to make it spike in at least half of 50 simulations) in the absence of inhibition varied from less than 20 to more than 100. At the population level, the activation curves of our simulations closely matched experimental data both with inhibition (control condition in Pouille et al., 2009) and without (GABA blocked or “gabazine” conditions in Pouille et al., 2009), reproducing the experimentally observed extension of the CA1PC dynamic range through FFI (Figure 2B). The Schaffer collateral input strength that recruited 95% of CA1PC with no inhibition was 0.27 relative to the same with “control” FFI, well matching the experimental value of 0.26 in the presence of gabazine (Pouille et al., 2009).

What are the distinct contributions of basket and bistratified cells to the CA1PC dynamic range extension? When only activating bistratified synapses while selectively blocking basket synapses in the simulation, FFI mostly affects the recruitment of relatively less excitable CA1PCs, i.e., those requiring activation of more excitatory synapses to fire (Figure 2C). In this context we define as “easily excitable” any CA1PC recruited by 30 excitatory synapses or less in absence of any inhibitory inputs. Correspondingly, CA1PCs requiring more than 30 synapses to be recruited are considered “less excitable”. The activation curve in the Gabazine condition (Figure 2C) suggests a continuum of CA1PCs excitability without sharp separation between two groups. In general, less excitable cells tended to possess lower input resistance, but other factors may also play a role, such as the total number of branches or the specific branching patterns in each neuronal morphology.

In contrast, FFI by basket synapses without bistratified synapses affected the whole CA1PC population, more closely resembling the effect of the control condition (i.e., combined basket and bistratified). Hence, taken as individual neuronal populations, basket cells play a larger role in regulating the CA1PC dynamic range by FFI compared to bistratified cells. What features of basket cells enable them to regulate the CA1PC dynamic range more efficiently? Aside from their spike timing and activation curves, basket and bistratified cells also differ in the number and spatial distribution of their synapses onto CA1PCs. Although in our model the synaptic domains of basket and bistratified synapses are completely segregated on CA1 PC somato-dendritic domains, some degree of spatial overlap between these interneurons may indeed exist in real biological systems. To ascertain the effects of partial overlaps, we ran simulations corresponding to the extreme case in which all synapses (disregarding their spatial location on the dendritic tree) were set with basket-like (or bistratified-like) activation (Figure 2C). Specifically, to differentiate the effects of spike timing and of the activation curves in basket and bistratified cells from the number and spatial location of their synapses, we left the number and spatial distribution of all synapses intact, but we adopted for all synapses the spike timing and activation curve of one of the two interneuron types (Figure 2C dotted lines). In these conditions, the ability of bistratified cells to extend the dynamic range of CA1PCs increased dramatically, becoming more pronounced than that of basket cells or of the control condition (basket and bistratified combined). This result suggests that the differential FFI regulation of CA1PC activity by distinct GABAergic interneurons results from a combination of their specific biophysical and morphological properties. However, when the microcircuit details are computationally equalized, the spike timing and activation characteristics of bistratified cells are more conducive to extending the CA1PC dynamic range than those of basket cells.

These results shed light on the possible properties of the basket and bistratified interneurons responsible for the changes in the CA1pc I/O curve. Specifically, the activation curves of the two interneurons play a major role (empty blue and green symbols in Figure 2C). At the same time, the spatial distribution of the synapses also seems to significantly contribute to this effect: when the synapses of bistratified cells are moved perisomatically their effect changes from rather small (purple symbols) to highly prominent.

### Single CA1PCs Input/Output Curves

**Figure 3:**
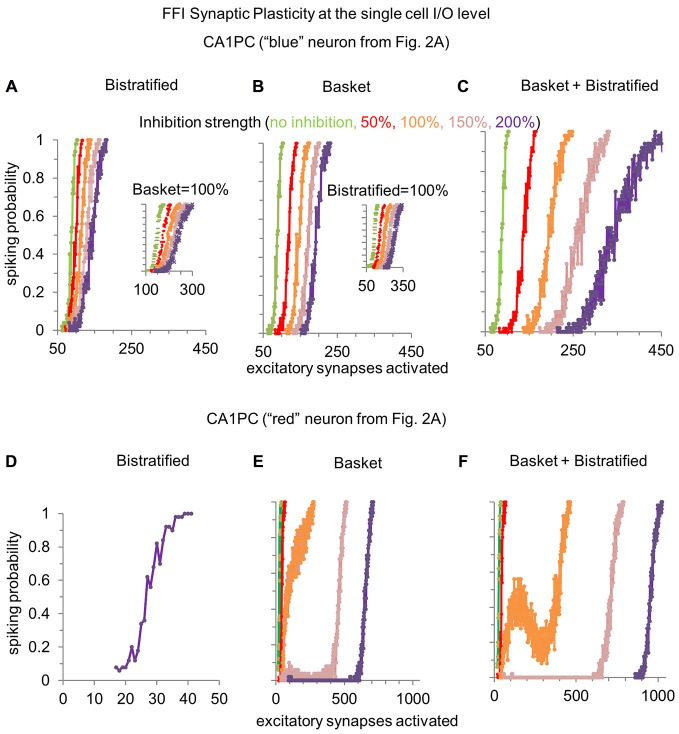
**Modulating the synaptic strength of basket and bistratified interneurons onto relatively less or more excitable CA1PCs. (A)** Increasing the strength of FFI by bistratified interneurons on a single CA1PC recruited by a high number of excitatory synapses progressively reduces the response gain. When compared to the no inhibition condition (in green), the computational operation performed by bistratified cells can be reduced to a division of the CA1PC I/O curve. Inset shows the same effect when basket cell synapses are activated. **(B)** Modulating the synaptic strength of basket interneurons on the same CA1PC increases the response threshold. When compared to the no inhibition condition (in green), the computational operation performed by basket cells can be reduced to a subtraction of the CA1PC I/O curve. Inset shows the same effect when bistratified cell synapses are activated. **(C)** Simultaneously increasing the strength of basket and bistratified FFI changes both the gain and threshold of CA1PC response to stimulation. **(D–F)** Same as **(A–C)** but on a CA1PC recruited by a low number of excitatory synapses. Note the emergence of a buffering effect with baseline inhibitory strength of combined basket and bistratified FFI (orange curve of **F**).

Next we examined the effects of different levels of FFI on individual CA1PCs. Although pharmacological treatments allow to increase or decrease the overall post-synaptic consequences of GABAergic transmission (Ferrante et al., 2008), computational simulations enable the selective manipulation of individual interneuron types. Thus, we modeled the progressive increase (150% and 200%) and decrease (50% and 0%) of FFI by bistratified cells alone, basket cell alone, and basket and bistratified combined (Figure 3), illustrating the results on CA1PC input/output curves using the same neurons singled out in Figure 2A.

Modulating the synaptic strength of bistratified interneurons alone onto a relatively less excitable CA1PC (one requiring more excitatory synapses to spike) changed the slope of its input/output curve (Figure 3A). This corresponds to a reduction of response gain with increasing FFI. In other words, bistratified cells enable CA1PCs to perform a divisive operation on their I/O curve. In contrast, altering the synaptic strength of basket cells regulated the intercept of the CA1PC input/output. This corresponds to a rise of the response threshold with increasing FFI (Figure 3B). Functionally, this translates in basket cells enabling CA1PCs to perform a subtractive operation on their I/O relationship. These complementary effects of bistratified and basket cells on CA1PCs remained present when the other interneuron class was also activated at its baseline strength (insets in Figures 3A,B). The two effects could be combined by varying at the same time the synaptic strength of both basket and bistratified cells (Figure 3C), controlling simultaneously slope and intercept.

When instead considering a relatively more excitable CA1PC, bistratified interneurons alone displayed no effect whatsoever on the input/output curve (Figure 3D). This result is explained by the stronger input required to activate bistratified cells (Figure 1D). The modulation of basket cell synaptic strength onto easily excitable CA1PCs led to the emergence of a plateau in the input/output curve, corresponding to a signal processing buffer (Ferrante et al., 2009). When basket and bistratified cells acted together, their combined effect synergistically enhanced the buffering effect, occasionally producing a “reversal” input/output zone (Figure 3F), even though bistratified interneurons alone did not alter CA1PC activity (Figure 3D).

### FFI Buffering of the CA1PC Input-Output Relation

**Figure 4:**
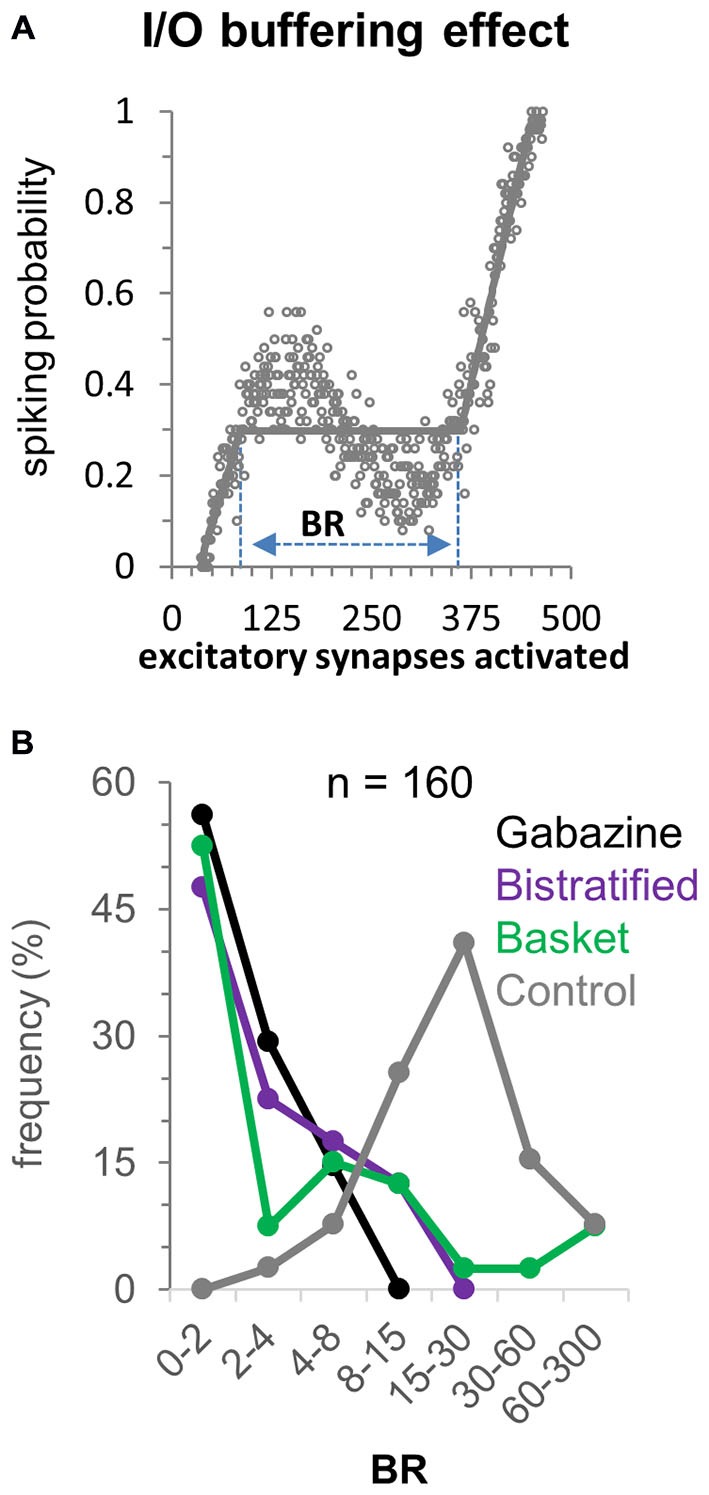
**Buffering effect of FFI on the CA1PC input/output relationship. (A)** Computation of the buffering range (BR) following the definition of Ferrante et al., 2009. **(B)** Frequency of BR values over the 40 CA1PC morphologies with no inhibition, basket alone, bistratified alone, and combined basket and bistratified FFI (all at baseline range). The synergistic FFI effect of basket and bistratified interneurons generates BR values between 15 and 30 synapses in approximately half of the neurons.

To quantify the distinct contributions of different FFI pathways to the input/output buffering of CA1PCs, we measured the buffering range (BR) of activated excitatory synapses within which the response remains constant or decreases (Ferrante et al., 2009). This is achieved by fitting the computed spiking probability with three lines (the second of which of zero slope) by minimizing the squared distance from all data points in the 50 simulations (Figure 4A; simulation noise is due to the spatial redistribution of activated synapses along the dendritic tree). In the gabazine (Figure 4B; no inhibition) condition most (56%) CA1PCs do not exhibit input/output buffering, and the few exceptions (due to synaptic redistribution noise) are confined to limited buffering ranges (<8 synapses). Basket and bistratified FFI, each taken in isolation, produced modest to moderate buffering effect, with e.g., ~10–20% of CA1PC buffered for more than eight activated excitatory synapses. In contrast, the combined FFI by both basket and bistratified interneurons produced substantial buffering, with the vast majority of CA1PCs showing a buffering range of 10–100 activated excitatory synapses. This result suggests that FFI buffering of CA1PCs synergistically produces the greatest computational impact through the interaction of diverse interneuron populations.

### Distinct and Synergistic Effects of FFI on CA1PC Spike Timing

**Figure 5:**
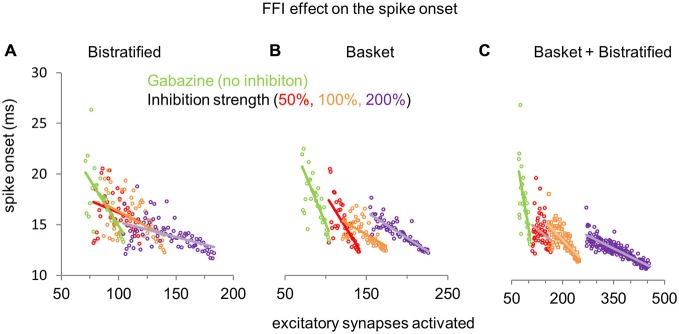
**Effect of FFI on CA1PC spike onset. (A)** Modulation of CA1PC spike onset by increasing synaptic strength of bistratified interneurons alone. **(B)** Same as **(A)**, but altering the synaptic strength of basket interneurons alone. **(C)** Same as **(A,B)**, except simultaneously modifying the synaptic strengths of both basket and bistratified interneurons. The gabazine data (green dots and line: no inhibition) are the same for all conditions and are repeated across panels for reference.

How does the modulation of FFI in basket and bistratified interneurons, alone or combined, affect the spike onset of CA1PCs? In the absence of inhibition (gabazine condition), activation of larger numbers of excitatory synapses generally reduces the CA1PC spike onset. Progressively increasing the synaptic strength of bistratified interneurons alone (Figure 5A), while requiring a correspondingly growing number of activated excitatory synapses to elicit a spike, also reduced the spread in the spike onset of CA1PCs across the entire stimulation range. Specifically, bistratified FFI nearly halved the spike onset differential from 13 ms with no inhibition (26.3 ms at 76 EPSPs minus 13.3 ms at 109 EPSPs), to 7.1 ms at 200% inhibitory strength (18.7 ms at 116 EPSPs minus 11.6 ms at 182 EPSPs). Modulating the synaptic strength of basket cells produced a slightly greater reduction of the spike onset differential relative to bistratified interneurons, down to 5.2 ms (17.2 ms at 156 EPSPs minus 12 ms at 226 EPSPs), but also created a sharper separation in the number of activated excitatory synapses required to spike (Figure 5B). The combination of basket and bistratified interneurons reduced the CA1PC spike onset differential similarly to the basket cells alone (5.7 ms), but synergistically produced the largest increase in the number of activated excitatory synapses necessary to fire a spike (Figure 5C). Overall, the effect of FFI on CA1PC firing is to reduce sensitivity (increase the number of required inputs) while reducing temporal delay and jitter. As a consequence, potentiating FFI synapses may allow CA1PCs to respond to heightened excitation with improved temporal fidelity.

## Discussion

Seminal experiments have demonstrated a fundamental role of FFI on CA1PCs in enforcing temporal fidelity (Pouille and Scanziani, 2001) and in expanding the dynamic range, in terms of both spiking probability of single neurons and fraction of recruited cells at the population level (Pouille et al., 2009). However, it has so far remained challenging to distinguish experimentally the specific contributions of distinct FFI pathways across the diversity of GABAergic interneurons. In addition to providing insights on possible circuit mechanisms, quantifying the separate effect of multiple FFI pathways is important in light of the state-dependent rhythmic change in CA1PCs excitability due to the temporal redistribution of inhibition over perisomatic and dendritic domains (Somogyi et al., 2013).

Furthermore, previous experimental studies only compared CA1PC activity with and without inhibition, and could not investigate the consequence of gradually modulating or potentiating FFI. This effect could be important because inhibitory synaptic plasticity regulates CA1PCs spiking (Saraga et al., 2008) and feedforward disinhibition mediates hippocampal long-term potentiation in CA1PCs (Ormond and Woodin, 2009). In order to bypass existing experimental limitations, we pursued a computational modeling strategy to explore a broader range of possible mechanisms regarding the interaction between fast-spiking basket cells, regular-spiking bistratified cells, and CA1PCs.

Our simulation analyses suggest that basket cells are overall more effective than bistratified cells in expanding the dynamic range of CA1PCs (Figure 2C). This differential influence is likely due to the number and distributions of the respective synapses, since adopting the timing and activation response of bistratified cells for all synapses augmented the effect of FFI. Furthermore, when activated alone, bistratified interneurons mostly affect less excitable CA1PCs, i.e., those recruited by a high number of excitatory synapses. However, when basket cells are also activated, the bistratified FFI exercises a clear effect on more excitable CA1PCs as well.

The ability to gradually and independently alter the synaptic strength of basket and bistratified cells while keeping the other interneuron type constant or silent also revealed a double-dissociation of the effects of these two FFI pathways on the response properties of CA1PCs. Specifically, regulating basket cell synapses affected the input/output intercept or spiking threshold of CA1PCs, while altering bistratified cell synapses modulated the CA1PC input/output slope or spiking probability gain. This result is important because it suggests that basket and bistratified cells perform two functionally distinct operations into the I/O of CA1PCs: basket cells subtract, while bistratified divide the sigmoidal I/O of CA1PCs. The ability of simulations to isolate the independent effects of the interneuron activation curves, axonal distributions, and synaptic properties may in future work help determine the biophysical determinants of these complementary transformations.

We previously showed that FFI in the dentate gyrus buffered the input/output curve of principal (granule) cells, with the buffering range and buffered firing rate modulated by the number and weight of incoming excitatory synapses on the inhibitory interneuron (Ferrante et al., 2009). Recent experimental evidence lends support to this mechanism (cf. red curve of Figure 9D in Sun et al., 2014), and direct experimental testing in the same neuronal circuit now appears feasible (Li et al., 2013). Similarly, in this study, FFI buffering was observed when the CA1PC activation curve overlapped substantially with those of the inhibitory interneurons, most notably when both basket and bistratified cells were activated at their baseline level. Why, in these same conditions, was CA1PC input/output buffering not observed by Pouille et al., 2009? The most likely reason is that the input strength was not finely controlled at the single cell level in that study: for each set of recordings in every CA1PC, only one or two data points were acquired in the range of input strengths across which the spike probability went from 0 and 1 (i.e., where the plateaus or response reversal would be found), preventing the detection of any possible buffering effect. Synaptic input strength (Perez-Rosello et al., 2011) and its temporal summation (Migliore et al., 2004) can be highly regulated/shaped by intrinsic neuronal properties. It would be interesting to test how intrinsic cellular differences affect the dividing, subtracting, and buffering I/O operations performed by the different types of FFIs in CA1PCs.

Our spike onset results suggest that, notwithstanding specific differences between basket and bistratified interneurons, potentiating FFI synapses could maintain CA1PC spike timing constant for larger input strength. This might be considered as an additional and complementary aspect of the broad phenomena related to homeostatic plasticity (Turrigiano, 1999). These interactions are also likely to influence network dynamics over time through multiple parallel mechanisms. For example, GABA_A_-mediated FFI modulates hippocampal spike timing-dependent plasticity (Jang and Kwag, 2012). FFI also underlies the propagation into CA1 of cholinergically induced gamma oscillations intrinsically generated in CA3 (Zemankovics et al., 2013), but not the intrinsic generation of faster gamma oscillations in CA1 (Craig and McBain, 2015). Interestingly, recent *in vitro* and *in vivo* results (Shay et al., 2015; Tsuno et al., 2015) suggest that in neurons with strong *I*_h_ conductances, inhibitory synaptic inputs may enable post-inhibitory APs in restricted phases of theta oscillations. This alternative role of FFI could provide a possible mechanism to encode spatial navigation (Hasselmo, 2013). Such *I*_h_-dependent post-inhibitory rebound spiking could be dynamically unmasked by plastic regulation of *I*_KA_ (Ascoli et al., 2010).

Despite the electrophysiological and morphological realism of our computational model, it is impractical if not impossible to capture the full range of variability observed in nature. For instance, experiments tend to be noisier (i.e., displaying larger variability) when compared to simulations (Figure 1B), probably due to cellular differences in biophysical properties not implemented in our model. It would be interesting to investigate how intrinsic differences in ionic channels and other membrane characteristics affect the FFI modulation of CA1PC input/output properties. Our model accounts for all excitatory synapses (Megías et al., 2001), thus including recurrent local feed-forward excitation by CA1 pyramidal cells. Possible biophysical differences between these recurrent CA1 synapses and the main input from CA3, including the ~2 ms delay due to di-synaptic activation, were not simulated. However, only ~10% of the excitatory synapses are from CA1 recurrent axons, thus these differential effects can be assume to be minimal.

In addition, this study focused on a specific sub-circuit of the CA1 network, namely, the FFI interaction of basket and bistratified cells onto CA1PCs. Nonetheless, the same interneurons also provide inhibitory feedback to CA1PCs (Ali et al., 1998), possibly enhancing FFI buffering during sustained CA1PC activity. More generally, a number of other GABAergic interneurons may also participate in the complex regulation of CA1PC response to CA3 pyramidal neuron input (Somogyi, 2010), including ivy cells, axo-axonic cells, trilaminar cells, quadrilaminar cells, Schaffer collateral-associated cells, apical-targeting cells, and oriens-alveus cells among others (see also Hippocampome.Org). Furthermore, the parallel, converging, and diverging interaction of these pathways can be coordinated by a diverse family of interneuron-specific interneurons (Francavilla et al., 2015). Given such complex circuitry, neurobiologically plausible models and detailed compartmental simulations can play an essential role in the elucidation of the computational mechanisms at play.

## Author Contributions

MF implemented the model, run the simulations, and analyzed the data. MF and GAA conceived the research design, interpreted the data, and wrote the manuscript.

## Conflict of Interest Statement

The authors declare that the research was conducted in the absence of any commercial or financial relationships that could be construed as a potential conflict of interest.

## Footnotes

^1^Hippocampome.Org

^2^senselab.med.yale.edu

## Abbreviations

FFI: Feedforward inhibition
CA1PCs: Pyramidal cells in area CA1 of the hippocampus
EPSP: Excitatory post-synaptic potential
IPSP: Inhibitory post-synaptic potential
sp: stratum pyramidale
sl-m: stratum lacunosum-moleculare
so: stratum oriens
sr: stratum radiatum.
